# Association between medical resources and the proportion of oldest-old in the Chinese population

**DOI:** 10.1186/s40779-021-00307-6

**Published:** 2021-02-16

**Authors:** Chao Tan, Cai-Zhi Tang, Xing-Shu Chen, Yong-Jun Luo

**Affiliations:** Department of Military Medical Geography, Army Medical Service Training Base, Army Medical University, Chongqing, 400038 China

**Keywords:** Longevity, GDP, Medical resource, API, Oldest-old

## Abstract

The potential association between medical resources and the proportion of oldest-old (90 years of age and above) in the Chinese population was examined, and we found that the higher proportion of oldest-old was associated with the higher number of beds in hospitals and health centers.

**Supplementary Information:**

The online version contains supplementary material available at 10.1186/s40779-021-00307-6.

## Background

Life expectancy is influenced by many factors, including social and economic development levels, environmental factors, lifestyle choices and genetics [1]. Past studies on longevity mostly focused on regional differences [2], and the influence of genes and the natural environment. Some of these studies did not consider the intrinsic interactions among the factors that could influence longevity. Therefore, specific aims of the current study include: 1) to analyze the spatial characteristics of the long-lived population (referred to as oldest-old) in China; 2) to estimate the distribution of the factors that influence longevity; and 3) to systematically and quantitatively analyze the influence of different factors on longevity and identify the key factors determining the distribution of the long-lived population.

## Materials and methods

### Data acquisition and preprocessing

#### Oldest-old population, hygiene and the economy

The oldest-old population, hygiene and economic data in the 31 provinces of China (except for Hong Kong, Macao and Taiwan) were downloaded from the National Bureau of Statistics [3]. Definition of rural (villages and towns), urban areas (cities), and the oldest-old population was defined as 90 years and above, and derived from the 6th National Population Census data of 2010.

Gross domestic product (GDP) data were obtained from the National Bureau of Statistics for 2011. These variables were standardized as follows: the proportion of the oldest-old per 100,000, GDP per person, and the number of beds in hospitals and health centers per 1000 persons. For multivariate regression, actual values of the variables, rather than the standardized values of the variables, were used since longevity is affected by the total GDP, the number of beds and Air pollution index (API).

#### Air quality

Air quality data were acquired from the China Environmental Protection Network [4]. Data from 86 cities in 2010 were available. API is a dimensionless index based on PM_10_, SO_2_ and NO_2_ to describe air quality and short-term trends, and is divided into 5 levels (Additional file 1: Tab.S1). Annual API level was calculated based on daily reports and interpolated using ArcGIS 10.2 (ESRI, Redlands, CA, USA).

### Data and statistical analysis

#### Spatial interpolation

Since API was available only from 86 cities in China, air quality data were interpolated using ArcGIS 10.2, inverse distance weight (IDW) interpolation.

#### Correlation analysis

The correlation between two variables was analyzed using the following Pearson equation (Formula 1) using SPSS 19 (Statistical Product and Service Solutions, IBM, Armonk, NY, USA):

1 $$ {r}_{xy}=\frac{\sum_{i=1}^n\left({x}_i-\overline{x}\right)\left({y}_i-\overline{y}\right)}{\sqrt{\sum_{i=1}^n{\left({x}_i-\overline{x}\right)}^2{\sum}_{i=1}^n{\left({y}_i-\overline{y}\right)}^2}} $$

where *x*_*i*_ and *y*_*i*_ represent the variables, $\overline{x}$ and $\overline{y}$ represent the average *x*_*i*_ and *y*_*i*_, and *r* is the correlation coefficient.

In addition to analysis using a zero-order model (not considering the potential impact of covariants), data were also analyzed using a second-order model (controlling the potential impact of two covariants).

#### Regression analysis

Multivariate linear regression analysis was conducted to examine the association between the proportion of oldest-old and factors. The criteria for entering independent variables into the equation was: Enter, Criteria = PIN (0.05) and Pout (0.1).

## Results

### Spatial characteristics

The proportion of oldest-old was higher in the eastern and central regions of China (Additional file 1: Fig. S1). Rural areas had a higher proportion of oldest-old than in towns and cities in 28 out of the 31 provinces (Additional file 1: Fig. S2). The proportion of oldest-old residing in rural areas and cities varied considerably (12.16–85.70%, 4.30–81.52% respectively), while the proportion in towns was 4.70–24.73%.

### Factors associated with the proportion of oldest-old

#### GDP

In general, GDP per capita was higher in the eastern regions than in the western regions. Shanghai has the highest GDP per capita (74,572.54 yuan) (Additional file 1: Fig. S3). Guizhou has the lowest GDP per capita (13,243.72 yuan).

#### The number of beds in hospitals and health centers

The number of beds in hospitals and health centers per 1000 persons was 2.33–6.80 (Additional file 1: Fig. S4). The number of beds in hospitals and health centers per 1000 persons in rural areas was 1.85–4.28. The number of beds in hospitals and health centers per 1000 persons in cities was higher than in rural areas, and varied considerably (3.00–10.89).

#### API

In general, annual API was lower in the southern regions than in the northern regions (Additional file 1: Fig. S5). The lowest was in Hainan. The highest API was in Gansu.

### Relationship between the proportion of oldest-old and influencing factors

The proportion of the oldest-old correlated positively with GDP (*r* = 0.876, *P* < 0.001, Additional file 1: Tab. S2), and the number of beds in hospitals and health centers (*r* = 0.905, *P* < 0.001). There was a trend for negative correlation between the proportion of the oldest-old and API, but statistical analysis failed to validate the finding (*r* = − 0.125, *P* = 0.502).

Due to the interaction among GDP and the number of beds in hospitals and health centers, we controlled the impact of covariants using second-order partial correlation analysis. The correlation coefficient between the proportion of oldest-old and the number of beds in hospitals and health centers is 0.633 (*P* < 0.001, Additional file 1: Tab.S3). The partial correlation coefficient between the proportion of oldest-old and the API is − 0.446 (*P* = 0.015).

The multivariate regression yielded the following equation: the proportion of oldest-old = 1.206 × GDP + 0.416× the number of beds in hospitals and health centers - 1161.246 × API + 67,387.873 (*F* = 60,882, *P* < 0.001, Additional file 1: Tab. S4). There was a statistically significant association between the proportion of oldest-old with the number of beds in hospitals and health centers (*P* < 0.001), API (*P* = 0.015), but not with GDP (*P* = 0.119, Additional file 1: Tab. S5).

## Discussion

In our analysis, the proportion of oldest-old correlated positively with the number of beds in hospitals and health centers, which in turn was correlated with GDP per capita. A 1% increase in income has been reported to be associated with 0.01% in mortality rate and ~ 0.02% increase in average life expectancy [5].

We failed to show a correlation between the proportion of oldest-old with API using a zero-order model. However, when using a second-order model to control GDP and the number of beds in hospitals and health centers, we noticed a negative correlation, implicating complex interaction among these factors. However, there is little evidence for an association between air quality and acute deaths [6].

The current study has several limitations. First, air quality was reflected only by API (that considers PM_10_ only), and not by PM_2.5_ due to data unavailability. More importantly, perhaps, separate API data for urban and rural areas were not available.

## Conclusions

The proportion of oldest-old in the population is higher in the eastern and central parts than the western part of China. In 28 of the 31 provinces, the proportion of oldest-old is higher in rural areas than in urban areas. Medical resources, as reflected by the number of beds in hospitals and health centers, is the most important factor that could increase longevity.

## Supplementary Information

**Additional file 1.**

## Data Availability

The dataset used and analyzed during the current study is available from the corresponding author upon reasonable request.

## Abbreviations

API: Air pollution index
GDP: Gross domestic product
IDW: Inverse distance weight
