# Supplementary material for: Association between medical resources and the proportion of oldest-old in the Chinese population
Source: Mil Med Res. 2021 Feb 16;8:14. doi: 10.1186/s40779-021-00307-6 (PMC7888075; doi:10.1186/s40779-021-00307-6)
Supplement: Supplementary file 1 — Additional file 1. [file 40779_2021_307_MOESM1_ESM.docx]

**Appendix Tab. S1** API range and air quality categories

| API | Air quality | Health effects | Proposed actions |
| --- | --- | --- | --- |
| 0-50 | Excellent | Little to no | Can move normally |
| 51-100 | Good | Little to no | Can move normally |
| 101-200 | Mild pollution | Symptoms of the susceptible population are slightly aggravated, and irritation symptoms appear in healthy people. | Physical and outdoor activities should be reduced in patients with heart disease and respiratory diseases. |
| 201-300 | Moderate pollution | Symptoms are markedly exacerbated in patients with heart and lung disease, exercise tolerance is reduced, and symptoms are common in healthy people. | Elderly people and patients with heart disease and lung disease should stay indoors and reduce physical activities. |
| >300 | Heavy pollution | The exercise tolerance of healthy people is reduced, with obvious strong symptoms and some diseases in advance. | Elderly people and patients should stay indoors to avoid physical exertion, and the general population should avoid outdoor activities. |

API. Air pollution index


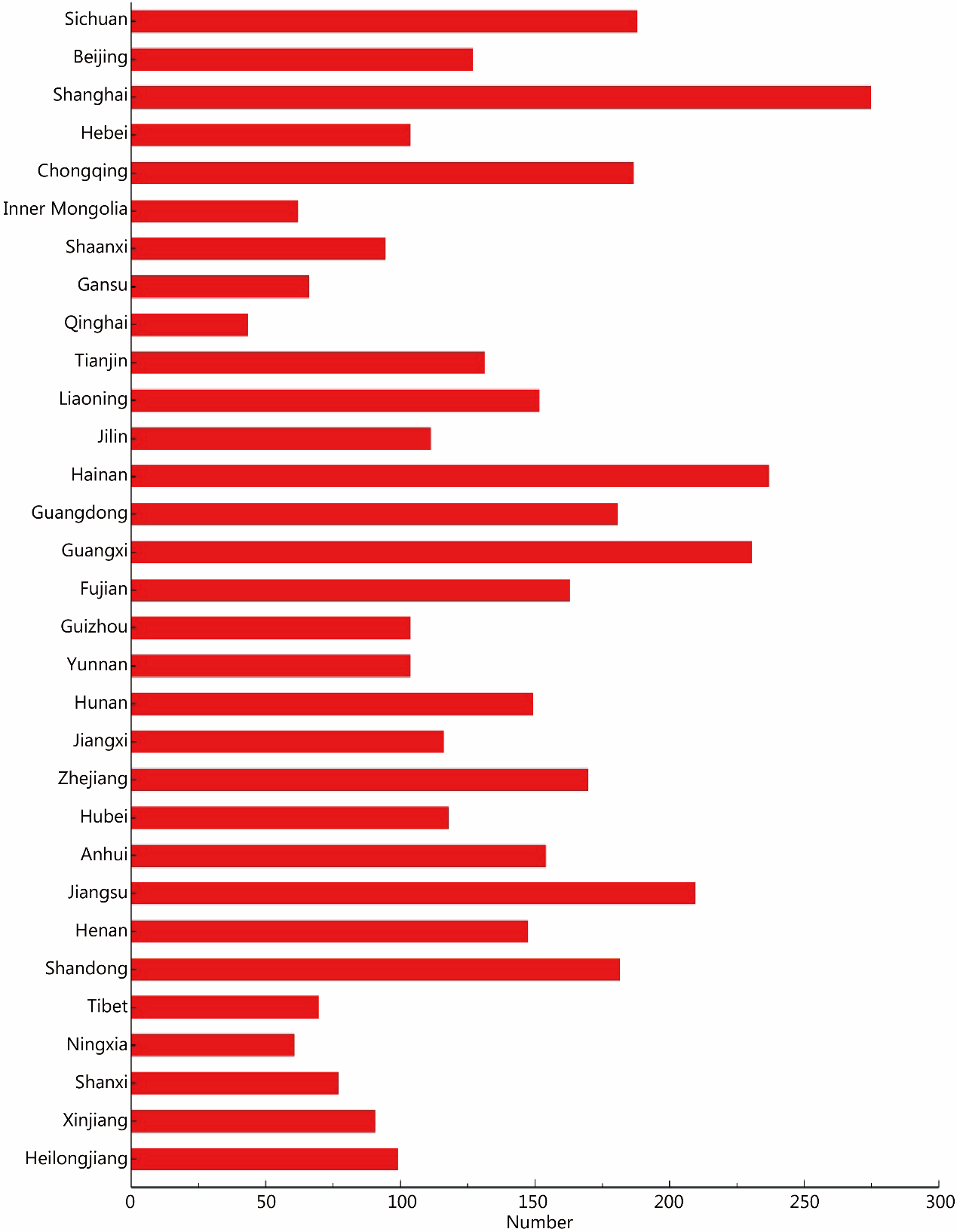


**Appendix Fig. S1** Number of individuals in the long-lived population per 100,000 persons in each province of China (except for Hong Kong, Macao and Taiwan)


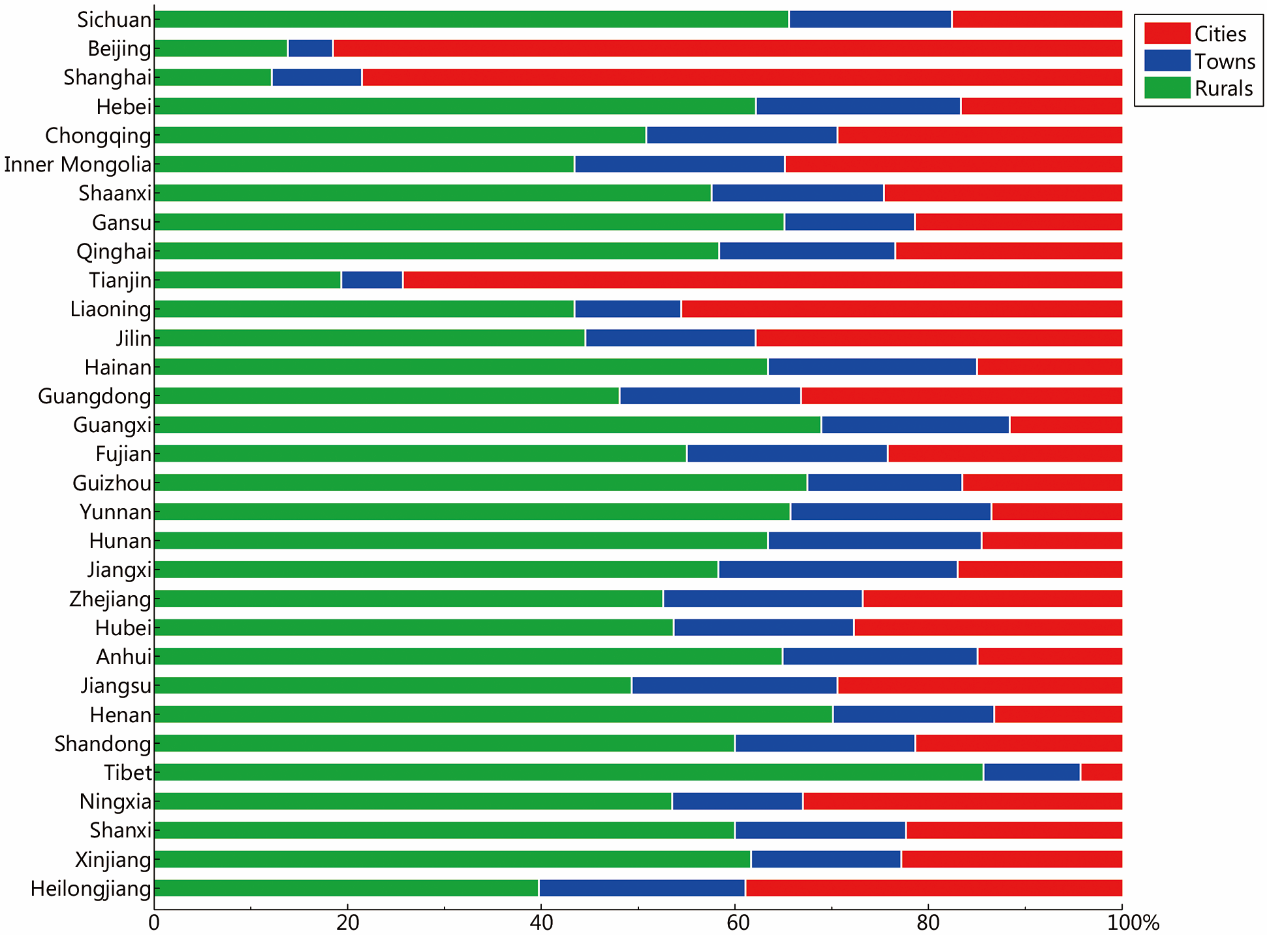


**Appendix Fig. S2** The proportion of the long-lived population of villages, towns and cities accounts for the long-lived population in the region


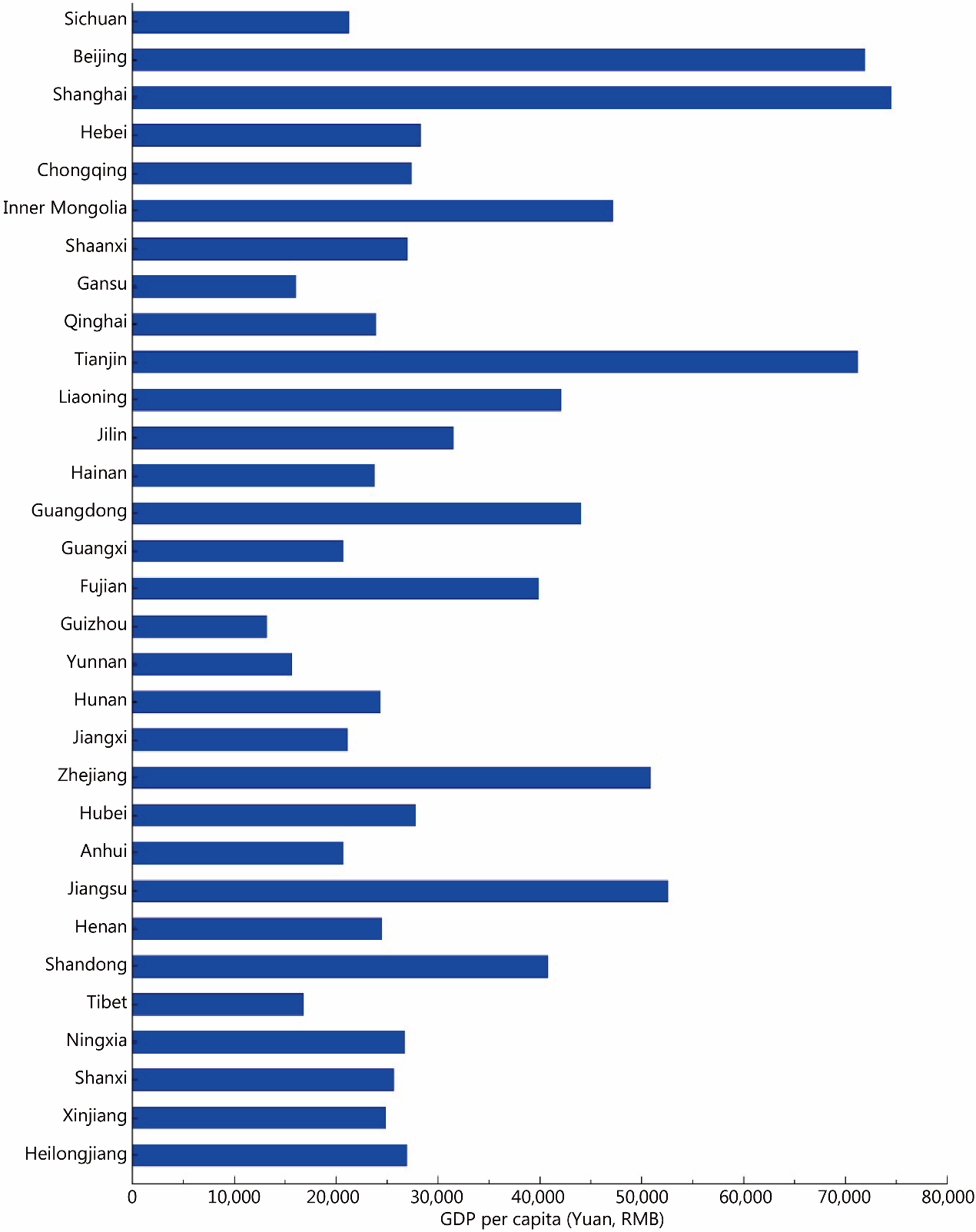


**Appendix Fig. S3** GDP per capita in each province of China (except for Hong Kong, Macao and Taiwan)


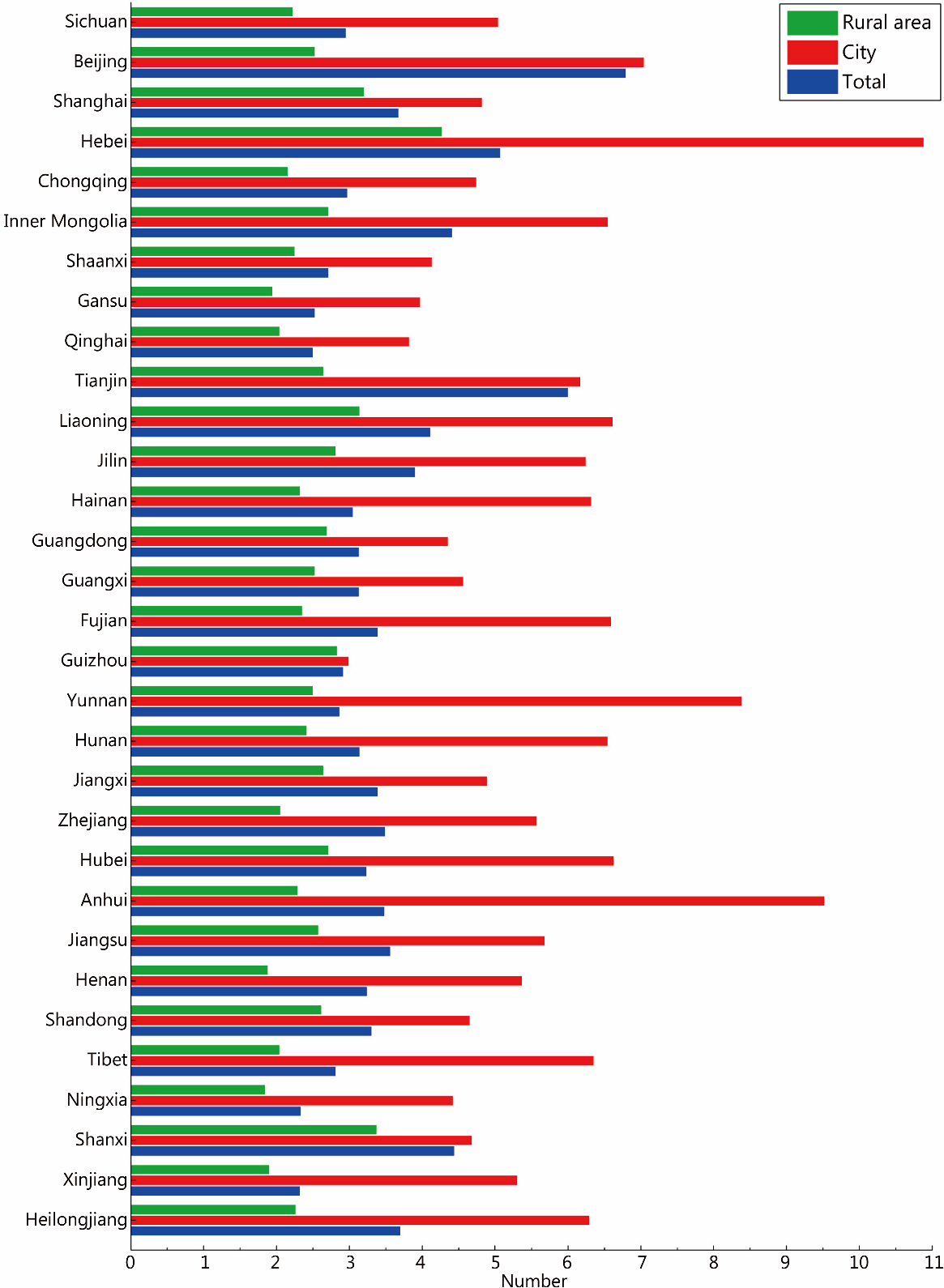


**Appendix Fig. S4** The number of beds in hospitals and health centers per 1000 persons in each province of China (except for Hong Kong, Macao and Taiwan)


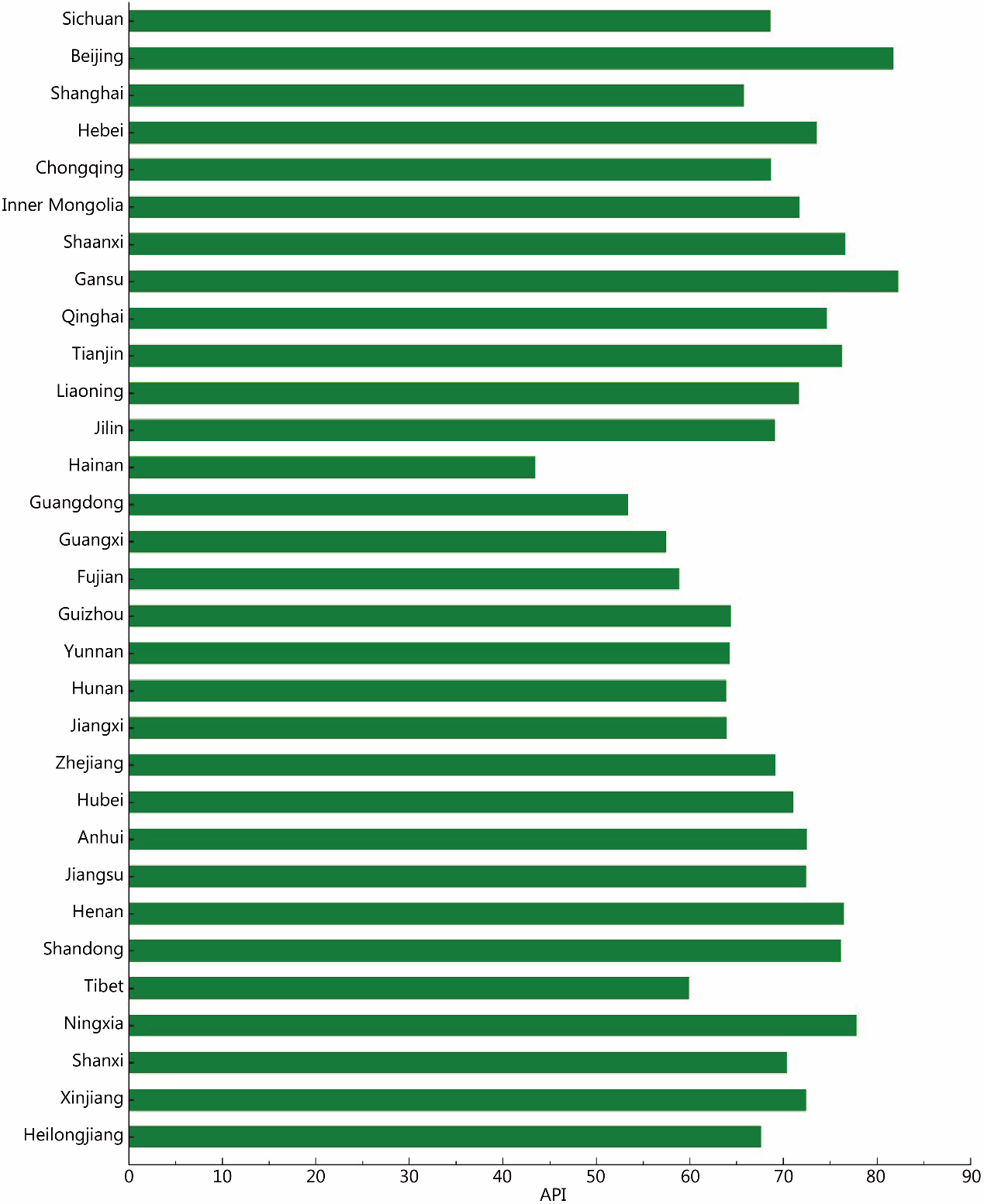


**Appendix Fig. S5** API in each province of China (except for Hong Kong, Macao and Taiwan)

**Appendix Tab. S2** The zero-order partial correlation of the long-lived population with GDP, beds and API

| Item | | Long-lived population | GDP | Beds | API |
| --- | --- | --- | --- | --- | --- |
| Long-lived population | Pearson correlation | 1 | 0.876^**^ | 0.905^**^ | -0.125 |
|  | Sig. (2-tailed) |  | 0.000 | 0.000 | 0.502 |
|  | *n* | 31 | 31 | 31 | 31 |
| GDP | Pearson correlation | .876^**^ | 1 | 0.901^**^ | 0.008 |
|  | Sig. (2-tailed) | .000 |  | 0.000 | 0.966 |
|  | *n* | 31 | 31 | 31 | 31 |
| Beds | Pearson correlation | 0.905^**^ | 0.901^**^ | 1 | 0.079 |
|  | Sig. (2-tailed) | 0.000 | 0.000 |  | 0.672 |
|  | *n* | 31 | 31 | 31 | 31 |
| API | Pearson correlation | -0.125 | 0.008 | 0 .079 | 1 |
|  | Sig. (2-tailed) | 0.502 | 0.966 | 0.672 |  |
|  | *n* | 31 | 31 | 31 | 31 |

^**^Correlation is significant at the 0.01 level (2-tailed); ^*^Correlation is significant at the 0.05 level (2-tailed). Beds present the numbers of beds in medical and health institutions

**Appendix Tab. S3** The second-order partial correlation of the long-lived population with GDP, beds and API

| Control variables | | | Long-lived population | API | GDP | Beds |
| --- | --- | --- | --- | --- | --- | --- |
| Long-lived population | | Correlation | 1.000 | -0.446 | 0.296 | 0.633 |
|  |  | Significance |  | 0.015 | 0.119 | 0.000 |
|  |  | df | 0 | 27 | 27 | 27 |
| GDP & Beds | API | Correlation | -0.446 | 1.000 | \ | \ |
|  |  | Significance | 0.015 |  |  |  |
|  |  | df | 27 | 0 |  |  |
| Beds & API | GDP | Correlation | 0.296 | \ | 1.000 | \ |
|  |  | Significance | 0.119 |  |  |  |
|  |  | df | 27 |  | 0 |  |
| API & GDP | Beds | Correlation | 0.633 | \ | \ | 1.000 |
|  |  | Significance | 0.000 |  |  |  |
|  |  | df | 27 |  |  | 0 |

Beds present the numbers of beds in medical and health institutions

**Appendix Tab. S4** ANOVA^b^ of the long-lived population with GDP, Beds and API

| Model | Sum of squares  (10^10^) | df | Mean square  (10^8^) | *F* | Sig. |
| --- | --- | --- | --- | --- | --- |
| Regression | 7.358 | 3 | 245.3 | 60.882 | 0.000^a^ |
| Residual | 1.088 | 27 | 4.029 |  |  |
| Total | 8.446 | 30 |  |  |  |

^a^Predictors: (Constant), beds, GDP, API; ^b^Dependent variable: Long-lived population

**Appendix Tab.S5** Coefficients^a^ of the long-lived population with GDP, Beds and API

| Model | Unstandardized coefficients | | Standardized coefficients | *t* | Sig. |
| --- | --- | --- | --- | --- | --- |
|  | *β* | Std. Error | *β* |  |  |
| Constant | 67387.873 | 30994.952 |  | 2.174 | 0.039 |
| API | -1161.246 | 447.862 | -0.182 | -2.593 | 0.015 |
| GDP | 1.206 | 0.750 | 0.259 | 1.609 | 0.119 |
| Beds | 0.416 | 0.098 | 0.686 | 4.246 | 0.000 |

Beds present the numbers of beds in medical and health institutions
